# *Toxoplasma gondii* seroprevalence among pregnant women attending antenatal clinic in Northern Tanzania

**DOI:** 10.1186/s41182-018-0122-9

**Published:** 2018-11-19

**Authors:** Eliakimu Paul, Ireen Kiwelu, Blandina Mmbaga, Rebeka Nazareth, Elias Sabuni, Athanasia Maro, Arnold Ndaro, Jo E. B. Halliday, Jaffu Chilongola

**Affiliations:** 1 Kilimanjaro Christian Medical University College, P.O. Box 2240, Moshi, Tanzania; 2 Kilimanjaro Clinical Research Institute, P.O. Box 2236, Moshi, Tanzania; 3 Kilimanjaro Christian Medical Center, P.O. Box 3010, Moshi, Tanzania; 4 Boyd Orr Center for Population and Ecosystem Health, Institute of Biodiversity, Animal Health and Comparative Medicine, College of Medical, Veterinary and Life Sciences, University of Glasgow, Glasgow, G12 8QQ UK

**Keywords:** *Toxoplasma gondii*, Toxoplasmosis, Serology, Molecular diagnostics, ELISA, PCR, Pregnant women, Tanzania, Zoonoses

## Abstract

**Background:**

Acute *Toxoplasma gondii* infection during pregnancy represents a risk for congenital disease, especially among women without previous exposure to infection. There is, however, a paucity of information about the epidemiology of *T. gondii* infection in pregnant women in Tanzania. This study aimed to determine the seroprevalence of *T. gondii* infection and associated demographic, clinical, and behavioral risk factors in pregnant women attending ante-natal clinic (ANC) at Kilimanjaro Christian Medical Center (KCMC), a referral medical center in Northern Tanzania.

**Methods:**

A hospital-based cross-sectional study was carried out from 1 February to 30 April 2017. Data on maternal demographic characteristics, obstetric history, knowledge, and practices related to *T. gondii* infection were collected from 254 pregnant women attending antenatal care at KCMC. A sample of 4 mL of blood was collected from each participant and sera prepared from each sample. Serum samples were tested for the presence of specific *T. gondii* IgG and IgM antibodies by indirect Enzyme-Linked Immunosorbent Assay (ELISA). DNA was extracted from whole blood for polymerase chain reaction (PCR) testing, targeting the DNA sequence coding for the Internal Transcribed Spacer 1 (ITS1).

**Results:**

The overall *T. gondii* seroprevalence, including both IgM- and IgG-positive individuals, was 44.5%. Of the 254 tested women, 102 and 23 were seropositive for *T. gondii*-specific IgG and IgM antibodies respectively and 113 individuals had antibodies of either or both classes. All IgM-positive samples were also tested by PCR, and all were negative. The majority (90%) of the women surveyed had never heard about toxoplasmosis. Consumption of raw vegetables [aOR = 0. 344; 95% CI 0.151–0.784; *p* = 0.011] and having regular contact with soil [aOR = 0.482; 95% CI 0.268–0.8681; *p* = 0.015] were both associated with *T. gondii* antibody status. Inverse relationships with probability of *T. gondii* exposure were observed, such that these practices were associated with reduced probability of antibody detection.

**Conclusion:**

Based on serology results, we report widespread exposure to *T. gondii* infection among pregnant women attending ANC in KCMC. The complex interaction of risk factors for *T. gondii* infection needs to be studied in larger longitudinal studies.

## Background

Toxoplasmosis is a globally distributed disease caused by *Toxoplasma gondii*, a protozoan parasite that belongs to the phylum Apicomplexa [1, 2]. It is estimated to infect more than one third of the world population and to be responsible for 1.2 million disability-adjusted life years (DALYs) annually [3]. *T. gondii* has a complex multi-host lifecycle. Humans are infected by this parasite mainly by ingesting food or water that is contaminated with oocysts shed by cats or by eating undercooked or raw meat containing tissue cysts [4, 5]. One of the major consequences of pregnant women becoming infected by *T. gondii* is vertical transmission to the fetus. Although rare, congenital toxoplasmosis can cause severe neurological or ocular disease (leading to blindness), as well as cardiac and cerebral anomalies in the newborn [6, 7]. Limitations in diagnostic techniques in less developed countries hinder the implementation of effective routine screening strategies.

The main diagnostic tests for *T. gondii* infection are serology for the detection of *T. gondii*-specific IgM and IgG antibodies, which indicate previous exposure. Confirmation of current infection is made by molecular tests, most commonly PCR [6, 8]. Maternal-fetal transmission usually occurs when the mother acquires a new infection just prior to or during pregnancy. The risk of infection to the fetus is low during early pregnancy but increases towards parturition. However, the earlier the fetus is infected, the greater the effects to the fetus and newborn [9]. This underscores the need for early diagnosis and appropriate treatment as the most reliable ways of reducing the risk of trans-placental transmission and subsequent sequelae in the newborn [10, 11]. Previous studies involving pregnant women attending antenatal clinics (ANC) indicate that the seroprevalence of *T. gondii* exposure in pregnant women varies substantially between and within countries. Data from Europe show a wide range of prevalence of *T. gondii* exposure in pregnant women across countries as measured by serology, with seroprevalence values from 9 to 67% [12–16].

In previous seroprevalence studies in Asian countries, the reported seroprevalence of *T. gondii* infection in pregnant women was 41.8% in India [6], 41% in Iran [16], and 49% in Malaysia [17]. In Africa, there have been relatively few studies of *T. gondii* prevalence. Seroprevalence values of 18.7% to 46.0% have been reported in Mozambique [18, 19], 30.0% in Benin [20], 40.8% in Nigeria [21], and 20.3% in Burkina Faso [22]. Previously identified risk factors for *T. gondii* infection include eating undercooked meat, warm and humid climates, drinking of unboiled contaminated water, and keeping a cat [5, 13, 23, 24]. There have only been a small number of previously published studies of toxoplasmosis in pregnant women in Tanzania [5, 25–28]. This cross-sectional study was conducted to determine the seroprevalence and identify risk factors for *T. gondii* exposure in pregnant women attending ANC at KCMC, in Moshi Town, Tanzania.

## Methods

### Study site and design

This was a cross-sectional, hospital-based study conducted in the period from 1 February to 30 April 2017 at the KCMC. KCMC is a tertiary university teaching hospital in Moshi town in Kilimanjaro region. It serves as a zonal referral hospital for five northern regions of Tanzania, serving about 15 million people from six regions: Tanga, Kilimanjaro, Arusha, Manyara, Singida, and Dodoma. As a referral medical center, it also serves patients from other regions of the country and neighboring countries. The major economic activities in the region include agriculture, horticulture, trading, tourism, and livestock keeping. Kilimanjaro region is characterized by a hot and wet climate, characteristics which have been documented to favor *T. gondii* oocyst survival. In addition, it is a tradition that residents of the region are heavy consumers of roasted meats (beef, chicken, and pork) [5, 29, 30], posing a risk for ingesting infective parasite tissue cysts from infected animals [4, 5, 13, 17].

### Sample size

The minimum sample size for seroprevalence determination was estimated using the Epi Tools online sample size calculator using the formula [*Z*2·(*p*)·(1 − *p*)]/*c*2, where *Z* = 1.96 for 95% confidence level (CI), *p* = expected true proportion of 20.0%, and *c* = minimal tolerable error at 95% CI (0.05). Computing with the above formula gives a minimum sample size of 246. This study enrolled 254 pregnant women.

### Study population

The study population for this study consisted of pregnant women who were 18 years old and above who were seeking routine antenatal care (ANC) at KCMC. The study excluded pregnant women who were below 18 years old, those who declined to participate, or those diagnosed with any other disease that needed immediate medical attention. Pregnant women who met study criteria were consecutively enrolled in the study until the desired sample size was reached.

### Data collection

Data on all study participants were obtained using a structured questionnaire through face to face interview (see SI file). The questionnaire used in this study was developed by investigators, with a few questions adopted from other studies [24]. Validity and reliability of the questionnaire were determined. It was first piloted on ten respondents before the actual study and these respondents were excluded during actual data collection and analysis. Validity and reliability were determined by using computer software IBM SPSS Version 20. In addition, two experts in the field of survey design approved the quality of questionnaire. After the pre-test, adjustments in phrasing were made.

Information collected for every participant included demographic data, obstetrical information, knowledge about Toxoplasmosis, and practices considered likely to be associated with *T. gondii* infection such as residential place (urban versus rural), education level, regular exposure to soil, owning cat, source of water, and consumption of raw vegetables. All obstetrical information gathered by questionnaire was also confirmed from medical files for accuracy for which prior consent was sought from participating mothers. Patients with mismatching information (in questionnaire and medical files) were excluded from the study. Before interviews, the questionnaire was pre-tested with 10 pregnant women at Mawenzi Regional Referral Hospital and Majengo dispensary to test the validity and relevance of questions. No personal identifiers were included in data collection forms.

### Blood sample collection and storage

For each consenting participant, 4 mL of venous blood was collected using a sterile disposable syringe. Two milliliters of each sample was placed into a serum separator tube (BD Vacutainer®, NJ, USA) and EDTA coated vacutainer tube respectively. Serum separator tubes were left at room temperature for about 10 min before serum was separated by centrifugation at 2000×*g* for 10 min in a refrigerated centrifuge and serum stored at − 20 °C before serological tests. Whole blood samples were stored at 4 °C until DNA extraction for PCR testing.

### Detection of *T. gondii-*specific IgM and IgG antibody by Enzyme-Linked Immunosorbent Assay (ELISA)

Detection of *T. gondii-*specific IgM and IgG was performed using commercial EUROIMMUN ELISA kits (EUROIMMUN®, Lübeck, Germany) as per the manufacturer’s instructions. The absorbance value for each sample and control samples was obtained using a bichromatic spectrophotometer (BioTek®, CA, USA) at 450 nm. Results were defined semi-quantitatively by calculating a ratio of the extinction value for test samples (optical density) to the extinction value of the calibrator positive and negative control sera. Cut points used were < 0.8 for negative, ≥ 0.8 to < 1.1 for equivocal, and ≥ 1.1 for positive result for both *T. gondii*-specific IgG and IgM ELISA as per the manufacturer’s instructions. Seropositivity was reported if samples were positive for *T. gondii*-specific IgM or IgG positive or both.

### *Toxoplasma gondii* polymerase chain reaction (PCR)

#### DNA extraction

DNA was extracted from samples collected from participants who were *T. gondii*-specific IgM positive by ELISA. Total DNA was extracted from whole blood stored with EDTA using the DNeasy Blood & Tissue kits (Qiagen, Valencia, CA). Two hundred microliters of whole blood was lysed with Proteinase K in QIAamp Buffer AL. The purified DNA was then eluted in 200 μL Buffer AE following the manufacturer’s instructions.

#### Nested PCR

The PCR amplified DNA sequence coding for the Internal Transcribed Spacer 1 (ITS1), following a previously described protocol with slight modifications [28]. The PCR assays were performed using a Tetrad2 Peltier thermocycler (BIO-RAD). Briefly, the primers used in the first round of the PCR (external primers) were as previously described [31]. The forward primer (NN1) sequence was 5′-TCAACCTTTGAATCCCAA-3′, and the reverse primer (NN2) sequence was 5′-CGAGCCAAGACATCCATT-3′. During the first round, 2 μL of the sample DNA extract was mixed with 10 μl of 2× master mix (Promega, Madison, WI, USA) a premixed PCR amplification buffer, 1 μl of each external primer (NN1 And NN2), and 6 μl of reagent grade H_2_O making a final reaction volume of 20 μl. Thermal amplification conditions were 5 min at 95 °C followed by 35 cycles of denaturation at 95 °C for 1 min, annealing at 55 °C for 1 min and extension at 72 °C for 1 min with an elongation step of 5 min at 72 °C.

For the second round PCR, 2 μl of the first-round PCR product, diluted to 1:5 with molecular grade water (Qiagen, Valencia, CA) was used as the template for the second-round PCR in a total volume of 20 μl under the same PCR conditions as in the first round, using the internal primers NNP1 forward primer (5′-GTGATAGTATCGAAAGGTAT-3′) and NP2 reverse primer (5′ACTCTCTCTCAAATGTTCCT-3′) as previously described. [32]. Positive controls (2 μL *T. gondii* DNA) and negative controls (2 μL reagent grade H_2_O) were included in each PCR run. The amplified products were visualized by electrophoresis using 2% agarose gel in Tris-Borate-EDTA (TBE) buffer (Promega, Madison, USA) stained with 0.05% ethidium bromide for visual detection by Ultraviolet Transilluminator (E-BOX, Vilber Lournar France). PCR positivity for *T. gondii* was defined by the presence of a 300-bp band on visualized in the gel.

#### Statistical analysis

Data were analyzed using SPSS v.22 (IBM® Corp., Armonk, NY, USA). Descriptive data were reported as frequencies, means, and medians. Categorical data are reported as tabulation of proportions. Logistic regression analyses were used to examine associations between *T. gondii* seropositivity (positive for IgM and/or IgG vs negative for both) and candidate predictor variables. Bivariable models were constructed for all candidate demographic, behavioral, and awareness variables evaluated. All variables with a coefficient *p* value ≤ 0.2 in the bivariable model were considered in the multivariable modeling [33]. Crude odds ratios (cORs) from bivariable models and adjusted odds ratios (aORs) from the multivariable model are reported (Table 3).

## Results

### Demographic data

Samples and linked data were collected from 254 women over a period of 60 days starting on 1 February 2017. Among the 254 women who took part in this study, the mean age in years was 29.9 years (SD ± 5.5) (Table 1). The majority of participants, 158/254 (62.2%), resided in Moshi Urban district in Kilimanjaro region. Most participating women (74.8%) were educated beyond primary school. The majority of the women (80.0%) were employed and doing business/traders, with smaller proportions being students, housewives and peasants (Table 1). Of the 254 pregnant women included in the study, 174 (68.5%) had one or more previous conceptions and 178 (70.1%) were in their third trimester of gestation at the time of this survey. Results of HIV status from participants’ files showed that 13 of 242 participants with known HIV status (5.3%) were HIV positive. With regard to knowledge of toxoplasmosis, 227 of the 254 women (89.4%) reported that they had not heard about toxoplasmosis and 91.3% reported that they did not know about its mode of transmission (Table 1).

**Table 1:** Participant characteristics (*n* = 254)

Variables	Frequency (*n*)	Percentage (%)
Age group (years)		
< 25	43	16.9
25–35	171	67.3
> 35	40	15.7
District of residence		
Moshi Rural District	96	37.8
Moshi Urban District	158	62.2
Education level		
Primary education	64	25.2
Secondary education	77	30.3
College/pre university education	45	17.7
University education	68	26.8
Occupation		
Student	17	6.7
House wife	19	7.5
Farmer	15	5.9
Employed	91	35.8
Self-employed business women	112	44.1
Gravidity index		
Primigravida	80	31.5
Multigravida	174	68.5
Trimester		
1st trimester	10	3.9
2nd trimester	66	26.0
3rd trimester	178	70.1
HIV status		
Positive	13	5.1
Negative	229	90.2
Not known	12	4.7
Heard about toxoplasmosis		
Yes	27	10.6
No	227	89.4
Knowledge on toxoplasmosis routes of infection		
Yes	22	8.7
No	232	91.3
Knowledge on effect of toxoplasmosis during pregnancy		
Yes	14	5.5
No	240	94.5

### Seroprevalence of *T. gondii*-specific IgG and IgM antibodies

A total of 254 serum samples were tested for *T. gondii*-specific IgG and IgM antibodies. Serum samples from 113 women were seropositive for *T. gondii*-specific IgG and/or IgM, giving an overall seroprevalence of 44.45%. Of all tested samples, 102 (40.2%) and 23 (9.1%) were seropositive for *T. gondii*-specific IgG and IgM antibodies, respectively. Ten participants (3.9%) were positive for *T. gondii*-specific IgM but negative for *T. gondii*-specific IgG and 77 (30.3%) participants were positive for anti-*T. gondii* IgG but negative for *T. gondii*-specific IgM (Table 2).

**Table 2:** Seropositivity of *T. gondii*-specific IgG and IgM antibodies

IgG positivity	IgM positivity			
	IgM +ve*	IgM –ve	IgM borderline	Totals
IgG +ve*	12 (4.7)	77 (30.3)	13 (5.1)	102 (40.2%)
IgG –ve	10 (3.9)	131 (51.6) **	3 (1.2) ***	144 (56.7%)
IgG borderline	1 (0.4)	7 (2.8) ***		8 (3.1%)
Totals	23 (9.1%)	215 (84.6%)	16 (6.3%)	254

Numbers indicate counts with respective percentages in parentheses. *All samples in the row and column are positive (*n* = 113). **Negative (*n* = 131). ***Equivocal (*n* = 10)

### Factors associated with *T. gondii* seropositivity

In bivariable analysis, reported consumption of raw vegetables, regular soil contact, occupation, and education level were associated with *T. gondii* seropositivity (Table 3). In multivariable analysis, consumption of raw vegetables and regular soil contact adjusted for participant’s occupation, education, and HIV status were significantly associated with *T. gondii* exposure. Participants who reported consumption of raw vegetables were less likely to be seropositive for *T. gondii* antibodies compared to those who were not consuming raw vegetables [aOR = 0.344; 95%CI 0.151–0.784; *p* = 0.011]. The probability of exposure of participants who had regular soil contact was lower than in those who did not report regular contact with soil [aOR = 0.482; 95%CI 0.268–0.8681; *p* = 0.015].

**Table 3:** Logistic regression analyses of participant factors associated with *T. gondii* seropositivity

Variable	Category	Positive: *n*/*N* (%)	Bivariable			Multivariable		
			cOR	95% CI	*p* value	aOR	95%CI	*p* value
Age groups	< 25	20/43 (46.5)	1					
	25–35	72/171 (42.1)	0.836	0.427–1.637	0.602			
	> 35	21/40 (52.5)	1.271	0.536–3.012	0.586			
Residence	Rural	42/96 (43.8)	1					
	Urban	71/158 (44.9)	1.049	0.630–1.749	0.854			
Education level	Primary Education	33/64 (51.6)	1.431	0.806–2.541	0.221	1.478	0.727–3.005	0.281
	≥ Secondary education	80/190 (42.1)	1			1		
Occupation	Student	5/17 (29.4)	1			1		
	House wife	7/19 (36.8)	1.400	0.346–5.672	0.637	0.991	0.215–4.571	0.991
	Famers	8/15 (53.3)	2.743	0.640–11.753	0.174	1.917	0.390–9.425	0.423
	Employed	43/91 (47.3)	2.150	0.700–6.599	0.181	1.511	0.441–5.174	0.511
	Business	50/112 (44.6)	1.935	0.639–5.860	0.243	1.742	0.542–5.595	0.351
HIV status	Negative	100/229 (43.7)	1			1		
	Positive	7/13 (53.8)	0.676	0.220–2.075	0.494	0.699	0.220–2.223	0.544
Cat ownership	Yes	20/39 (51.3)	1.355	0.684–2.683	0.384			
	No	93/254 (43.3)	1					
Eating undercooked meat	Yes	89/201 (44.3)	0.98	0.533–1.800	0.947			
	No	24/53 (45.3)	1					
Eating raw vegetables	Yes	92/221 (41.6)	0.415	0.195–0.886	0.023	0.344	0.151–0.784	0.011**
	No	21/33 (63.6)	1			1		
Eating unwashed fruits	Yes	20/44 (45.5)	1.028	0.535–1.975	0.933			
	No	93/210 (44.3)	1					
Water sources	Tap water	104/237 (43.8)	0.803	0.292–2.211	0.671			
	Others	9/17 (52.9)	1					
Regular soil contact	Yes	74/181 (40.9)	0.617	0.357–1.066	0.083	0.482	0.268–0.868	0.015**
	No	39/73 (53.4)	1			1		
Habit of eating soil (geophage)	Yes	20/49 (40.8)	0.788	0.433–1.434	0.435			
	No	93/205 (45.4)	1					

*cOR* crude odds ratio, *aOR* adjusted odds ratio

## Discussion

Toxoplasmosis is a parasitic infection of public health importance. Efforts to determine its sero-epidemiology, especially in high-risk groups such as pregnant women, are important to understand the distribution and level of exposure to this pathogen. These data can be useful for the design of toxoplasmosis control and prevention strategies. The current study was designed to determine the seroprevalence and associated risk factors for exposure to *T. gondii* among pregnant women in a Tanzanian referral hospital. We observed a seroprevalence of 40.2% based on *T. gondii*-specific IgG ELISA. This seroprevalence suggests endemicity of the parasitic infection in the studied area in line with previous reports [26, 34].

Serological detection of *T. gondii*-specific IgM is an indicator of recent or current infection [27]. In this study, 12 (4.7%) participants were positive for both *T. gondii*-specific IgM and IgG antibodies of which 10 (3.9%) were *T. gondii*-specific IgM positive but *T. gondii*-specific IgG negative. When the *T. gondii*-specific IgM-positive samples were subjected to PCR, none of them was positive for *T. gondii* infection. This finding highlights the challenges of comparing serological and molecular diagnostic test data and of obtaining samples that are appropriate for PCR-based detection of *T. gondii* DNA. *T. gondii*-specific IgM test is used by most laboratories to determine if a patient has been infected recently but *T. gondii*-specific IgM antibodies may remain detectable for prolonged periods of time beyond the acute infection [35]. It is not surprising therefore that positivity for *T. gondii*-specific IgM in this study did not correspond with the PCR results generated. We used peripheral blood samples in this study which are likely to have few circulating parasites [1, 7, 12]. In this regards, the diagnostic dilemma reported in this study underscores the usefulness of relevant samples such as amniotic fluid or placenta tissue for diagnosis of *T. gondii* infection among pregnant women. Studies show that although amniotic fluid or placenta samples might be more relevant for the diagnosis of toxoplasmosis by PCR, they have the disadvantage of additional sample processing steps before processing the diagnosis.

In this study, more than two thirds of the studied population had regular contact with soil (i.e., were involved in activities such as gardening and farming). Previous studies have reported regular soil contact increases the risk for *T. gondii* infection [36, 37]. However, our findings show that participants who had regular soil contact were less likely to be *T. gondii* seropositive, compared to those who had no regular soil contact. This observation contradicts with the frequent finding of soil contact being a risk factor for *T. gondii* infection. In this study, participating women were mostly urban dwellers, with a small proportion of the women participating in farming activities as their primary occupation. The extent and nature of soil contact among women living in town and those from rural areas could not be determined in this study.

The current study found that eating raw vegetables were associated with reduced probability of *T. gondii* seropositivity. However, this protective effect is inconsistent with that reported in Ethiopia [38] where consumption of raw vegetables was associated with increased risk of *T. gondii* infection. A possible explanation for our results has to do with eating habits and food preparation practices in the studied population. About half (44.9%) of participants in this study had access to clean tap water which reduces the likelihood for infection with *T. gondii*. Eighty percent of the participants had post-primary school education which suggests better understanding of hygiene principles. Similar to findings in this study, other studies have also reported the absence of associations between consumption of raw vegetables and *T. gondii* infection [39, 40].

Previous studies had reported that being rural dweller [41], having low education/illiterate [42], eating undercooked meat [43], cat ownership [39, 40], drinking water from public sources [38], and being HIV positive [44] were all found to be risk factors for contracting *T. gondii* infection. However, none of these factors had significant association with *T. gondii* seropositivity in this study. This may be explained by the fact that this was a hospital-based study, involving a special group of pregnant women, who may not represent the wider community. There is a need to conduct more studies that involve pregnant women from different settings to establish and compare the *T. gondii* seroprevalence among these different groups. There is also a need to conduct longitudinal studies across seasons of the year to determine the seasonality of toxoplasmosis in pregnancy.

## Conclusion

The current study reveals exposure to *T. gondii* infection and evidence of acute infection of *T. gondii* among pregnant women in the study area. Additionally, this study demonstrated low awareness of risk factors for toxoplasmosis. Soil contact and eating raw vegetables were the only factors associated with *T. gondii* exposure. Our study was limited by the small sample size and being a hospital-based cross-sectional study which could have missed some important epidemiological associations. However, our findings provide a useful piece of evidence of exposure to *T. gondii* among pregnant women.

## Abbreviations

ANC: ante natal clinic
ELISA: Enzyme-Linked Immunosorbent Assay
KCMC: Kilimanjaro Christian Medical Center
